# Comparative analysis of bariatric surgery outcomes and preoperative body composition in individuals with obesity with and without binge-eating disorder: a retrospective study

**DOI:** 10.1186/s40337-026-01628-4

**Published:** 2026-05-09

**Authors:** Xinping Wang, Dafang Zhan, Jie Zhang, Han Wang, Xiaoqin Pei, Miao Chen, Yuanchuan Zhang

**Affiliations:** 1 Department of General Surgery, The Third People’s Hospital of Chengdu, The Affiliated Hospital of Southwest Jiao Tong University, Chengdu, 610031 China; 2 Department of General Surgery, The Affiliated Hospital of Southwest Medical University, Luzhou, 646000 China; 3 Department of Healthcare-associated Infection Management, The Third People’s Hospital of Chengdu, Chengdu, 610031 China

**Keywords:** Binge-eating disorder, Obesity, Body composition, Bariatric surgery

## Abstract

**Objective:**

To compare preoperative metabolic parameters and body composition in individuals with obesity with and without binge-eating disorder (BED), and to evaluate postoperative short-term weight-loss outcomes in these two groups in the absence of structured preoperative cognitive-behavioral therapy (CBT).

**Methods:**

This retrospective analysis included 302 participants with obesity from the Western China Bariatric Surgery Cohort. Participants were classified into the BED group and the group without BED based on the Binge Eating Scale (BES) questionnaire and Diagnostic and Statistical Manual of Mental Disorders, Fifth Edition (DSM-5) diagnostic criteria. Basal metabolic parameters were assessed via an InBody 770 body composition analyzer, and rigorous follow-up tracking of postoperative weight variations was performed.

**Results:**

A total of 302 individuals with obesity were included, with 151 participants in the BED group and 151 in the group without BED. The proportion of females was significantly higher in the BED group than in the group without BED (*p* = 0.023). After adjustment for sex, there were no significant between-group differences in preoperative glucose, triglyceride, or total cholesterol levels. Sex hormone levels were comparable between groups in both males and females. Analysis of body composition showed no group differences in overall body weight, BMI, visceral fat area, or basal metabolic rate; however, females with BED exhibited greater leg fat mass (*p* = 0.037), while a trend toward a larger thigh circumference was observed among males (*p* = 0.050). In the linear mixed-effects model adjusted for sex and baseline weight, neither the main effect of group nor the group × time interaction was statistically significant, indicating comparable postoperative weight trajectories between the BED and NBED groups from baseline to 2 years after surgery.

**Conclusion:**

Individuals with obesity with and without BED showed largely comparable body-composition and metabolic characteristics. Bariatric surgery was associated with similar short-term weight-loss outcomes in individuals with obesity with and without BED, even in the absence of structured preoperative cognitive-behavioral therapy. These findings suggest that bariatric surgery may be an effective short-term weight-loss intervention for individuals with obesity and binge-eating disorder.

## Abstract (plain language summary)

Many people seeking weight-loss surgery also struggle with binge-eating disorder (BED), characterized by recurring episodes of eating large quantities of food. It has long been a concern whether patients with BED might have different metabolic health issues or see poorer results after surgery compared to those without the disorder. In this study, we compared 302 patients from a surgical cohort to see if having BED affected their physical health or their weight-loss success over a two-year period. Our results showed that, before surgery, patients with and without BED had very similar body compositions and metabolic health, such as blood sugar and cholesterol levels. More importantly, we found that both groups achieved nearly identical weight-loss results up to two years after their operations, even without receiving specific psychological counseling beforehand. These findings are encouraging because they suggest that weight-loss surgery is an equally effective tool for individuals with binge-eating disorder, helping them achieve successful short-term weight loss regardless of their eating patterns prior to the procedure.

## Introduction

Binge‑eating disorder (BED) was formally recognized in the Diagnostic and Statistical Manual of Mental Disorders, Fifth Edition (DSM‑5) in 2013 as an independent diagnostic entity [35]. It is one of the most prevalent eating-disorder subtypes worldwide [17]. The defining feature of BED is recurrent, uncontrolled episodes of excessive food intake accompanied by marked distress, and it is strongly associated with obesity [20]. Epidemiological studies indicate that the global pooled prevalence of BED is approximately 0.9%, with higher prevalence estimates reported in studies using DSM-5 diagnostic criteria (pooled estimate 1.3%, with values up to 2.3%), and substantially higher prevalence observed among individuals with obesity, with cohort studies reporting rates of approximately 14% [17, 29]. Previous studies have suggested that individuals with obesity and BED may exhibit a distinct metabolic and body-composition profile, including greater visceral adiposity, more severe insulin resistance, and chronic low‑grade inflammation [32].

Management of BED is typically multidisciplinary and may include psychological interventions, particularly cognitive-behavioral therapy (CBT), as well as pharmacological treatment when clinically indicated [21]. For candidates for metabolic and bariatric surgery (MBS), the American Society for Metabolic and Bariatric Surgery (ASMBS) recommends routine preoperative psychosocial evaluation to identify uncontrolled psychiatric conditions and implement behavioral interventions aimed at improving surgical outcomes [16]. However, previous studies have not reached a consistent conclusion on whether preoperative CBT provides additional benefits for postoperative weight outcomes [34, 36]. Several studies have found no significant advantage of preoperative CBT compared with standard care in terms of postoperative weight loss or maintenance.

Although biological [4], psychological [13], and social [5] mechanisms have been described, findings on body‑composition characteristics (e.g., fat distribution, visceral adiposity) and their metabolic correlates in individuals with obesity and BED remain limited. Body-composition assessment provides information beyond BMI by characterizing fat mass, lean mass, and adipose-tissue distribution, and may add value to metabolic risk assessment and individualized evaluation [22, 31]. Therefore, the present study aimed to assess body-composition and metabolic characteristics in individuals with obesity and BED, and evaluated short-term weight-loss outcomes after metabolic and bariatric surgery in individuals with obesity and BED in the absence of preoperative CBT.

## Methods

This retrospective study was based on data from an existing cohort of individuals with obesity who completed the Binge Eating Scale (BES).

All participants were required to meet the following inclusion criteria: (A) body mass index (BMI) > 28 kg/m² (according to Chinese obesity criteria); (B) age between 18 and 60 years; (C) no prior use of psychiatric medication, or medication use lasting less than three days at a dosage below the standard therapeutic level; and (D) willingness to participate in the study and provision of written informed consent.

Exclusion criteria were: a history of severe physical or neurological illness; any current or past diagnosis of a mental disorder (with the exception of binge-eating disorder); a history of traumatic brain injury with loss of consciousness exceeding 10 min; dependence on psychoactive substances, including alcohol (excluding nicotine, caffeine, or occasional social drinking); intellectual disability; pregnancy or breastfeeding; being critically ill or having significant physical disabilities; membership in a minority ethnic group; and a history of endocrine or other hormonal regulatory disorders.

A total of 376 individuals with obesity who completed the BES questionnaire were included in the retrospective analysis. Based on BES screening and subsequent diagnostic evaluation, 190 individuals were classified into the BED group, while 186 were classified into the NBED group. Participants with unavailable or incomplete body-composition analysis data were excluded, resulting in the exclusion of 39 individuals from the BED group and 35 individuals from the NBED group. After application of these exclusion criteria, a total of 302 participants remained eligible for analysis, with 151 participants in each group.

The assessment and grouping process consisted of two stages and was integrated into the routine preoperative clinical evaluation in our center. First, all eligible participants completed the BES. Participants with a BES score ≥ 18 underwent further evaluation by a psychiatrist using a structured diagnostic interview according to the DSM-5 criteria for BED. Participants who met the DSM-5 criteria were assigned to the BED group, while those who did not were assigned to the group without BED. Importantly, none of the participants received structured preoperative cognitive-behavioral therapy or other targeted BED-specific treatment prior to bariatric surgery.

### Body composition

Body composition measurements were performed by two researchers who had received standardized training in device operation and measurement protocols, including equipment principles, operational procedures, posture guidance, and data recording standards. Measurements were obtained using a direct segmental multi-frequency bioelectrical impedance analyzer (InBody 770), and the entire process strictly followed the manufacturer’s operational guidelines.

To minimize physiological and measurement variability, participants were instructed to follow standard preparation procedures, including short-term fasting, bladder emptying, wearing lightweight clothing, and removing all metal accessories. During the assessment, participants maintained a standardized standing posture to ensure consistency and accuracy.

The specific indicators measured included several key parameters reflecting body composition: body mass index (BMI), neck circumference, abdominal circumference, hip circumference, waist‒hip ratio, upper arm circumference, thigh circumference, total body water (TBW), protein, minerals, body fat mass (BFM), skeletal lean mass (SLM), fat-free mass (FFM), skeletal muscle mass (SMM), percentage of body fat (PBF), basal metabolic rate (BMR), visceral fat area (VFA), bone mineral content (BMC), the ratio of muscle mass to fat mass (MFR), and BMR/weight (BMR/W). Among them, MFR was calculated on the basis of the ratio of SMM to BFM, and BMR/W was calculated as the ratio of the BMR to body weight. These two indicators were calculated by researchers on the basis of the measurement data, whereas the values of the other indicators were automatically provided by the device’s electronic system, ensuring objectivity and standardization in the data acquisition process.

### Postoperative follow-up

Individuals with obesity included in this study were followed for two years after bariatric surgery. Weight data were collected at two time points, one year (± 1 week) and two years (± 1 week) after surgery, through outpatient visits, telephone calls, or text messages. For participants who were not able to attend in-person visits, postoperative weight was self-reported via telephone or text message. Information regarding postoperative psychological interventions, BED-specific treatments, or other non-surgical therapies was not systematically collected or evaluated during follow-up.

### Data analysis

Categorical variables were analyzed using the chi-square test or Fisher’s exact test. Given the significant between-group difference in sex distribution, preoperative metabolic indicators were further examined using sex-adjusted general linear models, with group and sex entered as fixed factors. Triglyceride values were natural log-transformed before modeling because of their skewed distribution. Baseline characteristics, body composition, and sex hormone levels were additionally compared using sex-stratified analyses, with independent-samples t-tests or Mann–Whitney U tests applied as appropriate. Postoperative weight trajectories were analyzed using a linear mixed-effects model, with group, time, sex, baseline weight, and the group × time interaction included as fixed effects and participant ID included as a random effect. A two-tailed p-value < 0.05 was considered statistically significant. All analyses were conducted using SPSS Statistics (Version 27; IBM Corp., Armonk, NY, USA).

## Results

### Basic characteristics and obesity-related indicators in individuals with and without BED

A total of 302 individuals with obesity were included in this study, with 151 assigned to the BED group and 151 to the group without BED. In this cohort, 50% of the individuals with obesity met the criteria for BED. There was a statistically significant difference in sex distribution between groups: the group without BED included 64 males (42.4%) and 87 females (57.6%), whereas the BED group included 45 males (29.8%) and 106 females (70.2%) (*p* = 0.023; Table 1).

**Table 1:** Basic information, obesity indicators and body composition in participants with BED or NBED

Variable	Male		Female	
	NBED BED	*p*	NBED BED	*p*
Sample	64 (42.4%) 45 (29.8%)		87 (57.6%) 106 (70.2%)	0.023^+^
Age(years)	35.4 ± 9.7 33.1 ± 8.3	0.197^#^	31(26,36) 30(25,35)	0.183^*^
Weight (kg)	119.4 ± 22.6 120.0(109.2,136.4)	0.342^*^	93.7(83.4,103.5) 97.2(86.9,107.5)	0.097^*^
BMI (kg/m^2^)	38.0(33.7,42.8) 39.2(37.0,43)	0.276^*^	35.8(32.6,39.1) 36.5(33.9,40.7)	0.114^*^
Neck circumference(cm)	44.1(41.8,47.1) 45.3(43.3,47.8)	0.114^*^	41.7 ± 3.4 42.4(39.8,44.7)	0.311^*^
Chest circumference (cm)	118.6 ± 7.3 121.1(116.1,124.2)	0.128^*^	109.5 ± 7.0 111.1 ± 7.2	0.129^#^
Abdominal circumference(cm)	126.6 ± 14.5 127.5 ± 10.6	0.281^#^	113.4 ± 7.2 114.7 ± 10.5	0.391^#^
Hip circumference(cm)	120.2(112.3,126.2) 121.8(115.8,125.7)	0.198^*^	112.8(107.7,117.8) 114.7 ± 7.0	0.078^*^
Waist - Hip ratio	1.06 ± 0.07 1.05(1.01,1.09)	0.642^*^	1.0 ± 0.05 1.0 ± 0.06	0.673^#^
Arm circumference (cm)	42.8(38.7,46.4) 42.7(41.2,46.9)	0.305^*^	39.0(36.2,41.3) 39.8(37.1,42.9)	0.148^*^
Thigh circumference(cm)	65.0 ± 5.0 67.0 ± 4.8	0.050^#^	61.5 ± 4.1 62.5 ± 4.2	0.108^#^
Total body water	51.1 ± 7.3 52.1 ± 6.8	0.511^#^	36.6(33.8,39.8) 37.1(34.4,40.9)	0.249^*^
Protein	13.7 ± 1.9 14.0 ± 1.2	0.435^#^	9.9 ± 1.2 10.1 ± 1.2	0.247^#^
Minerals	4.6 ± 0.7 4.7 ± 0.7	0.642^#^	3.4 ± 0.4 3.4 ± 0.4	0.266^#^
Body fat mass	48.7(37.3,60.3) 51.2 (41.8,58.0)	0.396^*^	44.4 ± 9.4 45.9 (39.2,53.0)	0.114^*^
Soft lean mass	65.7 ± 9.3 66.9 ± 8.7	0.496^#^	47.5 ± 5.9 50.5(46.7,59.4)	0.278^*^
Fat free mass	69.5 ± 10.0 70.8 ± 9.3	0.503^#^	50.2 ± 6.2 50.5(46.7,55.5)	0.279^*^
Skeletal muscle mass	39.3 ± 5.8 40.2 ± 5.5	0.449^#^	27.9 ± 3.7 28.5 ± 3.6	0.268^#^
Percent body fat	41.1 ± 6.1 42.1 ± 5.8	0.384^#^	46.6(43.9,50.6) 48.0(44.0,50.9)	0.298^*^
FFM of arm	4.2 ± 0.7 4.3 ± 0.6	0.700^#^	2.8 ± 0.4 2.8(2.6,3.1)	0.265^*^
FFM of trunk	31.7 ± 4.1 32.2 ± 3.9	0.597^#^	23.5 ± 2.8 23.9 ± 2.8	0.280^#^
FFM of leg	10.5 ± 1.6 10.9 ± 1.6	0.315^#^	7.6 ± 1.1 7.8 ± 1.0	0.520^#^
BFM of arm	4.7(3.1,7.4) 5.3 (3.7,6.7)	0.427^*^	4.1(3.4,5.4) 4.5 (3.5,5.9)	0.124^*^
BFM of trunk	24.4(19.6,27.8) 24.4 ± 3.6	0.700^*^	21.4 ± 3.6 22.0 ± 3.6	0.226^#^
BFM of leg	6.4(5.0,7.6) 6.6(5.5,7.5)	0.271^*^	6.2 ± 1.2 6.6 ± 1.4	0.037^#^
Basal metabolic rate	1871 ± 213 1898 ± 200	0.505^#^	1442(1361,1543) 1460(1380,1567)	0.402^*^
Visceral fat area	221.1(168.6,261.2) 218.5 ± 37.6	0.931^*^	201.1 ± 35.5 205.6 ± 33.8	0.270^#^
Bone mineral content	3.8 ± 0.6 3.9 ± 0.6	0.568^#^	2.8 ± 0.4 2.8 ± 0.4	0.369^#^
Muscle-fat ratio	0.84 ± 0.22 0.81 ± 0.20	0.374^#^	0.64(0.55,0.70) 0.60 (0.53,0.71)	0.289^*^
BMR per unit of body weight(BMR(kcal/day/kg))	15.9 ± 1.8 15.6 ± 1.6	0.289^#^	15.5 ± 1.3 15.1(14.1,16.2)	0.138^*^

*BMI* Body Mass Index, *BED* Binge eating disorder, *NBED* non-binge eating disorder, *FFM* fat-free mass, *BFM* body fat mass, *BMR* basal metabolic rate

The data are presented as the means ± standard deviations, medians (Q1, Q3), and n(%)

*Mann‒Whitney U test; #Independent t test; +Pearson χ² test

### Metabolic-related indicators and hormone levels in individuals with obesity with and without BED

After adjustment for sex, preoperative glucose, triglyceride (ln-transformed), and total cholesterol levels showed no significant between-group differences (all *p* > 0.05; Table 2). In both males and females, testosterone, progesterone, and estradiol levels did not differ significantly between the BED and NBED groups (all *p* > 0.05; Table 3).

**Table 2:** Blood glucose and lipid levels in participants with BED or NBED

variable	NBED		BED		*p*	Sex-adjusted *p*
	*n*	M (*P*_25_,*P*_75_)	*n*	M (*P*_25_,*P*_75_)		
Glucose	149	5.21 (4.67,6.07)	149	5.33 (4.78,6.35)	0.206^*^	0.057
Triglyceride	145	1.59 (1.21,2.44)	143	1.72 (1.14,2.56)	0.465^*^	0.169
Total cholesterol	145	4.88 (4.20,5.59)	143	4.98 (4.39,5.55)	0.290^*^	0.169

*BED* Binge eating disorder, *NBED* non-binge eating disorder

The data are presented as the means ± standard deviations or medians (Q_1,_ Q_3_)

*Mann‒Whitney U test; ^#^Independent t test

Sex-adjusted p values were obtained using general linear models with group and sex entered as fixed factors; triglyceride was analyzed after natural log transformation

**Table 3:** Hormone levels in participants with BED or NBED

variable	Male			Female		
	*n* NBED	*n* BED	*p*	*n* NBED	*n* BED	*p*
Testosterone	63 9.2 (7.2,14.4)	44 9.2 (6.9,12.2)	0.293^*^	81 1.8 (1.4,2.2)	101 1.5 (1.2,2.1)	0.141^*^
Progesterone	59 0.2 (0.1,0.2)	43 0.2 (0.1,0.2)	0.167^*^	78 0.2 (0.2,0.6)	98 0.2 (0.2,1.6)	0.762^*^
Estradiol	62 136 (108,171)	44 128 ± 47	0.320^*^	78 196 (142,407)	101 193 (141,339)	0.774^*^

*BED* Binge eating disorder, *NBED* non-binge eating disorder

The data are presented as the means ± standard deviations or medians (Q_1,_ Q_3_)

*Mann‒Whitney U test; ^#^Independent t test

### Body composition

There were no significant differences in overall body weight, BMI, visceral fat area, or basal metabolic rate between individuals with BED and those without BED. However, among females, individuals with BED had higher leg fat mass (*p* = 0.037), whereas among males, a trend toward a higher thigh circumference was observed (*p* = 0.050; Table 1).

### Postoperative body weight

Postoperative weight-related outcomes at 1 year and 2 years were comparable between the BED and NBED groups (Table 4). In the linear mixed-effects model adjusted for sex and baseline weight, the main effect of group was not statistically significant (*p* = 0.465), and the group × time interaction was also not significant (*p* = 0.446), indicating that postoperative weight trajectories from baseline to 2 years were comparable between the BED and NBED groups (Table 5). The main effect of time did not reach statistical significance (*p* = 0.068), whereas sex and baseline weight were significant covariates (both *p* < 0.001).

**Table 4:** Descriptive postoperative weight-loss outcomes in participants with BED or NBED

Variable	NBED		BED	
	*n*	M (*P*_25_,*P*_75_)	*n*	M (*P*_25_,*P*_75_)
1-year post-operative weight(kg)	128	70.0 (65.0,84.8)	126	70.0 (62.0,80.0)
ΔBMI at 1-year post-operative follow-up(kg/m^2^)	128	10.4 (8.6,13.7)	126	12.0 ± 4.43
ΔWeight at 1-year post-operative follow-Up(kg)	128	28.9 (22.9,38.4)	126	32.5 (24.1,40.6)
%EWL at 1-year follow-up	128	72.2 (58.9,88.8)	126	73.4 (62.8,91.1)
%TWL at 1-year follow-up	128	29.3 (23.7,35.0)	126	31.6 (24.9,37.8)
2-year post-operative weight(kg)	131	70.0 (64.0,83.0)	117	70.5 (62.0,83.5)
ΔBMI at 2-year post-operative follow-up(kg/m^2^)	131	10.0 (7.8,13.3)	117	11.5 (8.0,13.8)
ΔWeight at 2-year post-operative follow-up(kg)	131	27.6 (21.7,37.4)	117	31.2 (21.2,39.9)
%EWL at 2-year follow-up	131	71.8 (56.8,88.0)	117	72.1 (58.0,89.2)
%TWL at 2-year follow-up	131	33.0 (22.7,34.9)	117	29.9 (22.7,36.4)
ΔWeight (Y2-Y1)	123	0.3 (-2.0,3.1)	112	1.4 (-1.98,5.0)

*BED* Binge eating disorder, *NBED* non-binge eating disorder

Data are presented as the means ± standard deviations, medians (Q_1,_ Q_3_)

%EWL: percentage of excess weight loss = [(baseline weight - postoperative weight)/(baseline weight - ideal weight)] × 100%; BMI: Body Mass Index; ΔBMI = preop BMI - postop BMI; ΔWeight = preop weight - postop weight; Y1 = 1 year post-op; Y2 = 2 years post-op

**Table 5:** Linear mixed-effects model of postoperative weight

Effect	F	*p* value
Sex	11.162	< 0.001
Group	0.534	0.465
Time	3.359	0.068
Baseline weight	268.26	< 0.001
Group × Time	0.583	0.446

Linear mixed-effects model adjusted for sex and baseline weight, with group, time, and group × time interaction included as fixed effects

## Discussion

This study compared body-composition characteristics and postoperative weight-loss outcomes between individuals with obesity with and without BED. Body composition analysis revealed subtle differences in regional fat distribution between the two groups. During follow-up from baseline to 2 years after surgery, postoperative weight trajectories were comparable between individuals with obesity with and without BED. During the 2-year follow-up, BED was not associated with poorer postoperative weight-loss outcomes, even though patients did not receive structured preoperative psychological intervention.

The global pooled prevalence of binge-eating disorder in the general population is approximately 0.9%, with higher prevalence estimates reported in studies using DSM-5 diagnostic criteria [17]. In the present bariatric surgery cohort, 50% of individuals with obesity met the criteria for BED, a proportion higher than that reported in the general population and in pooled bariatric surgery samples. This difference may reflect differences in study population and assessment methods, as our cohort consisted of bariatric surgery candidates from a tertiary center and BED was identified using BES screening followed by psychiatrist-led DSM-5 diagnostic evaluation. Nonetheless, both previous studies and the present findings support the higher occurrence of BED among individuals with obesity, particularly those with severe obesity [12, 14, 41]. Multiple mechanisms may contribute to this association. Individuals with obesity often face dual pressures from both physiological and psychological factors, and binge eating may be perceived as a strategy to cope with stress. Various psychological factors (e.g., the need for emotional regulation and stress management) [18], social and environmental factors [2], and biological mechanisms (e.g., dopamine activity and addiction-related mechanisms) [4, 33] may all play significant roles in this relationship. At the same time, obesity itself may lead to increased food cravings and intake [23, 38], whereas BED may further exacerbate weight gain, forming a self-reinforcing cycle that worsens both conditions. This interaction not only affects patients’ physical health but also may have negative impacts on their mental health [19]. Therefore, a deeper understanding of the interplay between obesity and BED may help inform clinical assessment and management strategies.

This study revealed that the proportion of females was significantly higher in the BED group than in the group without BED, indicating a sex difference in the distribution of BED among individuals with obesity. Some studies have reported that the lifetime prevalence of binge eating disorder in females is approximately 1.9% [26], which is approximately twice that reported in males. This observation is consistent with prior literature reporting a higher prevalence of BED among females [37]. Sex differences in BED prevalence have been proposed to be influenced by multiple factors. Evidence suggests that estrogen may play a role in the regulation of appetite and metabolic processes [6, 7]. While BED is more frequently reported among females [25], the mechanisms underlying this sex difference remain unclear. Previous study suggests that affective and psychological characteristics commonly associated with BED, such as emotion regulation difficulties and negative affect, may play a role [30].

Although most adiposity-related indicators—including percentage body fat, total fat mass, and visceral fat area—did not differ significantly between groups, these measures were numerically higher in individuals with BED. Notably, females with BED had significantly greater leg fat mass (*p* = 0.037), and thigh circumference was numerically higher among males with BED (*p* = 0.050). These findings may suggest subtle differences in regional fat distribution between individuals with obesity with and without BED. Previous studies have shown that lower-body adipose tissue represents a distinct metabolic depot involved in lipid storage and energy buffering, and alterations in regional fat distribution have been discussed in relation to differences in energy storage capacity and metabolic characteristics [24, 28, 40]. However, the metabolic implications of greater thigh fat accumulation remain complex and context-dependent. Given the cross-sectional design of the present study and the absence of direct assessments of adipose tissue function or muscle fat infiltration, these interpretations remain speculative and cannot be confirmed by our data.

The present study showed that the presence of BED was not associated with poorer two-year postoperative weight outcomes. Descriptive analyses demonstrated comparable weight-related outcomes at both 1 and 2 years after surgery between the BED and NBED groups. More importantly, in the linear mixed-effects model adjusted for sex and baseline weight, neither the main effect of group nor the group × time interaction was statistically significant, indicating that postoperative weight trajectories were similar between groups from baseline to 2 years after surgery. These findings suggest that, within the current follow-up period, BED was not associated with inferior two-year bariatric surgery outcomes. This result is consistent with a previous study reporting no significant difference in postoperative weight loss outcomes between patients who did and did not receive preoperative CBT [34]. Although current guidelines recommend preoperative psychological interventions for bariatric surgery patients combined with BED to improve prognosis [11], none of the participants in the present study received structured preoperative CBT, and weight-loss outcomes at 1 and 2 years post-surgery did not differ significantly between the BED group and the group without BED [9]. Within the limits of our follow-up period, this finding suggests that the absence of formal preoperative CBT was not associated with clearly inferior short-term weight outcomes. At the same time, CBT for BED is typically delivered as a structured [21], multi-session intervention that often requires several weeks to months to complete [34, 39], whereas metabolic bariatric surgery has been demonstrated to be one of the most effective and durable treatments for severe obesity and its metabolic comorbidities [10, 16]. For individuals with obesity who have severe metabolic or cardiopulmonary comorbidities and clear surgical indications, a prolonged course of preoperative CBT could theoretically extend preoperative waiting times and delay access to surgery-related metabolic and cardiovascular benefits. However, because the present study did not systematically assess psychological symptoms or long-term mental health outcomes, these considerations require confirmation in prospective and randomized controlled studies. Therefore, decisions about preoperative CBT should be made on an individual basis within a multidisciplinary team, taking into account each patient’s physical risk, psychological needs, and urgency of treatment.

Although weight change from year 1 to year 2 was numerically greater in the BED group (1.4 kg vs. 0.3 kg), no significant between-group difference was observed within the current follow-up period, highlighting the importance of longer-term postoperative monitoring. Previous studies have shown that the five-year postoperative weight regain risk for individuals with obesity and BED is 18% greater than that for individuals with obesity without BED [27]. Such long-term weight trajectories have been linked to residual or recurrent binge-eating behaviors [1]. Although bariatric surgery is associated with favorable short-term weight outcomes in individuals with obesity and BED, differences in regional fat distribution and metabolic characteristics, such as relatively lower metabolic activity in thigh fat, have been discussed in the literature in relation to long-term weight regulation [3]. Accordingly, postoperative strategies including real-time monitoring of dietary intake and emotional fluctuations, regular assessment of body composition, targeted behavioral interventions [8, 36], and resistance training focused on the lower extremities to increase muscle mass may help support long-term weight maintenance [15].

### Limitations

This study has several limitations. First, because of its retrospective design, this study could only identify associations rather than establish causality. Second, the timeframe for assessing postoperative weight changes was relatively short, and the lack of comprehensive data on potential confounding factors—such as lifestyle, exercise, diet, and psychological interventions—may have introduced bias into the outcomes. In addition, follow-up completion appeared to be lower in the BED group at later postoperative time points, which may have introduced attrition bias. Although the linear mixed-effects model allowed inclusion of available repeated measures and partially mitigated bias due to missing data, the results should still be interpreted with caution.

Third, postoperative weight was collected via telephone or text message, meaning it was self-reported. This method is susceptible to recall and reporting biases, which may affect data accuracy. Fourth, randomized controlled trials are needed to further explore the impact of preoperative CBT on weight loss outcomes in individuals with obesity and binge-eating disorder. Fifth, potential mechanisms related to binge-eating disorder, including interactions between regional fat distribution and muscle function as well as the role of chronic low-grade inflammation in metabolic dysregulation, were not examined in depth in the present study and warrant further investigation through mechanistic research.

Future research should include larger sample sizes and encompass multicenter, multidimensional variables to construct a research framework for longitudinal observation and mechanistic exploration, aiming to help clarify the influencing factors of BED in weight loss and weight regain, thereby providing more reliable theoretical support for individualized treatment strategies.

## Conclusions and clinical significance

This study demonstrates that individuals with obesity and binge-eating disorder (BED) exhibit subtle differences in body composition, characterized primarily by a higher proportion of females and greater regional fat accumulation in the thigh region, while overall metabolic parameters and lean mass indices remain largely comparable to those of individuals with obesity without BED.

Importantly, the presence of BED was not associated with inferior two-year weight-loss outcomes following bariatric surgery. In sex- and baseline weight-adjusted analyses, postoperative weight trajectories from baseline to 2 years were comparable between individuals with obesity with and without BED. These findings indicate that bariatric surgery is associated with effective two-year weight reduction in individuals with obesity and BED under routine clinical practice conditions.

Overall, the present findings support the role of bariatric surgery as an effective weight loss treatment option for individuals with obesity and BED over a two-year period. Future prospective studies with longer follow-up and comprehensive evaluation of eating behaviors and psychological outcomes are warranted to further elucidate long-term weight trajectories and optimize multidisciplinary care in this population.

## Data Availability

The datasets used and/or analysed during the current study are available from the corresponding author on reasonable request.
